# The potential of UAV and very high-resolution satellite imagery for yellow and stem rust detection and phenotyping in Ethiopia

**DOI:** 10.1038/s41598-023-43770-y

**Published:** 2023-10-05

**Authors:** Gerald Blasch, Tadesse Anberbir, Tamirat Negash, Lidiya Tilahun, Fikrte Yirga Belayineh, Yoseph Alemayehu, Girma Mamo, David P. Hodson, Francelino A. Rodrigues

**Affiliations:** 1 International Maize and Wheat Improvement Center (CIMMYT), Addis Ababa, Ethiopia; 2 Ethiopian Institute of Agricultural Research (EIAR), Addis Ababa, Ethiopia; 3 Kulumsa Agricultural Research Center (KARC), Asella, Ethiopia; 4 International Maize and Wheat Improvement Center (CIMMYT), Texcoco, Mexico; 5 Lincoln Agritech Ltd, Lincoln University, Lincoln, New Zealand

**Keywords:** Biotic, Imaging and sensing

## Abstract

Very high (spatial and temporal) resolution satellite (VHRS) and high-resolution unmanned aerial vehicle (UAV) imagery provides the opportunity to develop new crop disease detection methods at early growth stages with utility for early warning systems. The capability of multispectral UAV, SkySat and Pleiades imagery as a high throughput phenotyping (HTP) and rapid disease detection tool for wheat rusts is assessed. In a randomized trial with and without fungicide control, six bread wheat varieties with differing rust resistance were monitored using UAV and VHRS. In total, 18 spectral features served as predictors for stem and yellow rust disease progression and associated yield loss. Several spectral features demonstrated strong predictive power for the detection of combined wheat rust diseases and the estimation of varieties’ response to disease stress and grain yield. Visible spectral (VIS) bands (Green, Red) were more useful at booting, shifting to VIS–NIR (near-infrared) vegetation indices (e.g., NDVI, RVI) at heading. The top-performing spectral features for disease progression and grain yield were the Red band and UAV-derived RVI and NDVI. Our findings provide valuable insight into the upscaling capability of multispectral sensors for disease detection, demonstrating the possibility of upscaling disease detection from plot to regional scales at early growth stages.

## Introduction

Globally, transboundary pathogens and pests are an increasing threat to crop production and food security. Increased trade and travel, coupled with a changing climate, are resulting in increased frequency and severity of crop disease outbreaks^1^. Of all the diseases that affect wheat, wheat rusts (stem/black rust (SR), stripe/yellow rust (YR) and leaf/brown rust (LR)) are among the most damaging, capable of causing epidemics on a vast scale with significant economic and production losses if the host plant-pathogen-climate relationship is conducive. Recent estimates indicate that global losses from wheat rusts equate to 15 million tonnes per year (2.9 billion USD)^2^. In Ethiopia, a major YR epidemic in 2010 affected an estimated 600,000 ha, resulting in production losses of 15–20% and causing economic losses on the order of 250 million USD^3,4^, and a severe SR epidemic in 2013/14 infected ~ 40,000 ha^5^. SR, which can cause 100% crop loss within weeks^6^, is re-emerging as a major concern, threatening up to 50 million ha of wheat (~ 25% of the world’s wheat area) in Asia and Africa^7^.

To mitigate the rust-related wheat production risk and reduce the spatial scale and frequency of rust epidemics, the cultivation of resistant wheat varieties is the most effective *ex ante* strategy. The application of fungicides is the most effective *ex post* strategy to reduce yield loss if resistance breaks down due to a new virulent race^4^. National and global breeding programs have been working over decades to develop and deploy rust-resistant wheat varieties, with increasing adoption in countries such as Ethiopia^8^. Rapid early-season detection, monitoring, and timely control of wheat rusts are critical to avoid large-scale outbreaks if susceptible varieties are being grown, especially in countries where fungicides are scarcely available or too costly for smallholders^9^.

Wheat rust infection can appear on any aboveground, living plant part in the form of visible yellow or reddish-brown pustules containing thousands of uredinio spores. YR caused by *Puccinia striiformis* f. sp. *tritici* occurs primarily on leaves and head, producing symptoms such as yellowing and necrosis of leaves, while SR caused by *Puccinia graminis* f. sp*. tritici* occurs primarily on stems (and to a minor degree on leaves and spikes), damaging the xylem tissue of the crop that conveys water and dissolved minerals from the roots to the rest of the plant. Subsequently, the reduced water and nutrient flow will weaken other plant parts, leading to necrosis of leaves. Thus, the spectral properties of the wheat canopy (e.g., pigmentation, moisture, and biomass) are altered under rust disease stress. Therefore, multispectral reflectance bands from optical UAV and satellite sensors and derived vegetation indices (VIs) related to crop growth (e.g., plant growth status, vegetation coverage, and pigmentation content) can be associated with crop susceptibility to diseases and consequently can be used for remote sensing (RS) application in wheat rust detection and monitoring^10–13^.

Through recent advances in sensor technology and data processing, unmanned aerial vehicles (UAVs) and very high-resolution satellite (VHRS; < 1 m spatial resolution) RS of plant canopies have been found to offer high potential for nonintrusive, extensive, rapid, and flexible measurements of plant biophysical (e.g., leaf area index; LAI) and biochemical (e.g., chlorophyll, carotenoids) properties at very high spatial and temporal scales.

To support wheat improvement breeding, UAV-based high-throughput phenotyping (HTP) has been recently investigated to assess plant growth development^14,15^, canopy architecture^16–18^, physiology^19,20^, reaction to abiotic stress^21–23^, crop disease and insect pest response^24–26^, and wheat yield^14,27,28^. Spectral and thermal measurements at the plant and canopy levels allow for monitoring the interactions between plant germplasm and environmental (abiotic and biotic) factors^29^. Recent literature reviews^30–34^ have described in detail UAV-HTP sensing systems, as well as their application potential, including specific phenotyping traits (e.g., VIs, LAI, biomass, and canopy height), advantages, and existing challenges.

Although satellite imagery was historically too coarse in terms of spatial and temporal resolution for phenotyping applications and crop trial monitoring^35^, novel multispectral VHRS systems and multi-satellite constellations (e.g., Pleiades, SkySat, WorldView series) can provide real-time to near-real-time coverage from sub-daily up to every 4 days with sub-meter to 2 m spatial resolution. Despite cloud cover issues, such VHRS imagery can serve as an effective and useful phenotyping tool, enabling automatic recording of isolated field trials across large geographical areas^36^. Recent research on wheat, maize and dry bean demonstrated strong and significant correlations between VIs extracted from UAV and VHRS imagery, confirming the feasibility of VHRS-HTP targeting biomass and yield^37–39^; however, such satellite applications for plant breeding programs are still scarce.

Compared to SR, YR symptoms are clearer to observe at the canopy level. Thus, the wheat rust research community has focused mainly on YR detection and monitoring through UAVs and satellites, testing a wide range of multispectral and hyperspectral sensing systems and techniques, including machine learning^40,41^. To the best of our knowledge, research on field phenotyping, monitoring or detecting SR in wheat using proximal or remotely sensed data has not been conducted thus far.

Satellite-focused studies have shown mono-temporal approaches based on one-stage spectral features suitable for YR mapping from field to regional scales^42–45^ but with the need that the single-phase RS dataset contains highly contrasting and distinct crop damage features at one specific crop growth stage^40^. For example, for several wheat diseases, such as powdery mildew, Fusarium head blight, and YR, the grain filling stage was identified as the appropriate timing for disease detection through RS data due to visible disease symptoms present on plant parts^12,42–49^. Using VHRS, the high potential of VIs such as NDVI and GNDVI derived from Quickbird imagery for quantifying wheat YR severity was demonstrated^42^. Moreover, WorldView imagery allowed for differentiating between wheat powdery mildew and YR diseases using both spectral bands and VIs^43^. For discriminating between healthy and YR-infected wheat at the regional scale, the spectral REDSI disease index applied to Sentinel-2 imagery showed an overall accuracy of 85.2% superior to nine commonly used VIs (< 81.5%)^44^. However, one-stage spectral features only reflect static host conditions at a single growth stage, displaying the crop disease situation as a snapshot at a certain point. The grain filling stage for wheat is relatively late in the cropping season, which in most cases is already too late for undertaking control measures and allows only for damage assessment. Although detection of rusts at an early stage is desirable, rust symptoms tend to be mild in early stages of infection and thus difficult to detect even with high-spatial-resolution and high-spectral-resolution images.

To address both strategies, the capability of multispectral high-resolution UAV and VHRS imagery as an HTP and rapid disease detection tool is assessed by analyzing the interaction between wheat varieties and rust diseases (here: YR and SR) in terms of early-stage detection and host plant response to rust and associated yield. The objectives were to (i) characterize the progressive development of YR and SR and associated grain yield loss using traditional visual disease estimations; (ii) assess the level of agreement of remotely sensed multispectral spectral features with visual disease scores of rust symptoms and their prediction capability to estimate visual disease scores at earlier growth stages (booting and heading), general disease progression, and grain yield; and finally, (iii) evaluate the potential to upscale UAV-based detection and phenotyping to very high-resolution spaceborne systems such as Pleiades and SkySat.

## Materials and methods

### Study site

**Figure 1:**
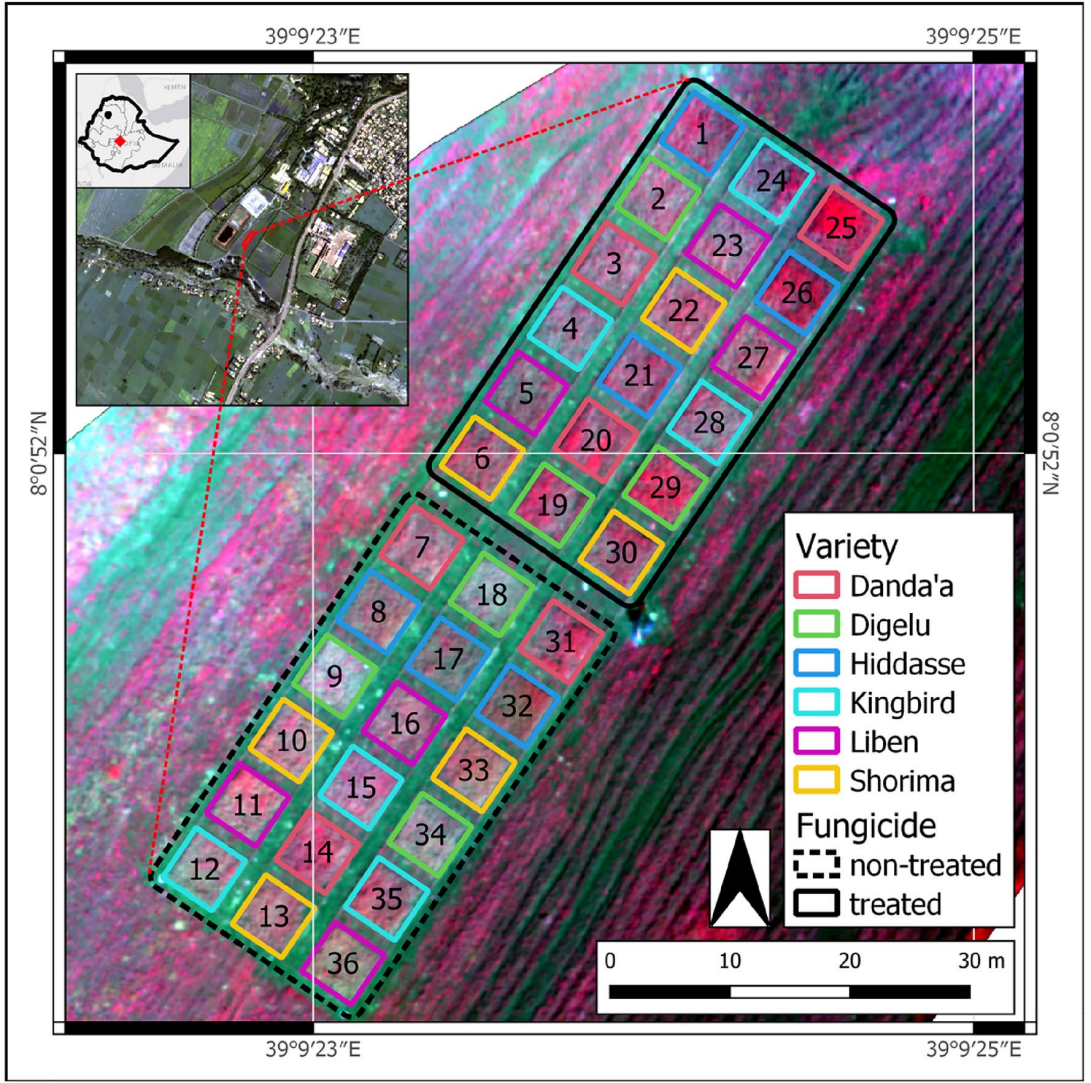
Location of the study site and experimental setup (experiment site: UAV false color composite using near infrared, red and green bands from 2020-10-29; heading stage / DAS 80; study site: Pleiades satellite scene from 2020-10-16). UAV imagery processed using Pix4Dmapper^52^ and figure prepared using QGIS version 3.14.16-PI^53^.

During the main cropping season in 2020, the field experiment was conducted at the EIAR Kulumsa Agricultural Research Center (KARC; 39° 10’ E/08° 02’ N; 2200 m above sea level), located in the Arsi zone (Tiyo district) of the Oromia Region, Central Ethiopia (Fig. 1). The mean annual rainfall is 820 mm with a unimodal distribution pattern, and the average air temperature ranges between 10.5 and 22.8 °C, allowing a growing period of 120–135 days^50^. The main cropping season is closely linked to the long rainy season (*Meher*; from June to September), with crops harvested between September and November. The predominant soil type is clayey soils (e.g., haplic vertisols, haplic luvisols)^51^. The climatic conditions are favorable for YR occurrence, and wheat fields located in the KARC area are prone to natural YR infection during the *Meher* season.

### Plant material

Six improved bread wheat varieties with varying resistance and reaction levels to SR and YR were selected for the experiment (Table 1). All varieties had been investigated for their agronomic performance and resistance to YR and/or SR in multiple locations in Ethiopia^8,54,55^. From the pool of available seed material, wheat varieties were chosen for each anticipated plant (host) reaction to current prevailing races of SR and YR: *resistant*, *moderately resistant/susceptible*, and *susceptible*. The plant material used in this experiment was procured from EIAR-KARC’s national wheat breeding program and plant pathology research division, complying with institutional and national guidelines and legislation.

**Table 1:** Characteristics of selected wheat varieties.

Variety	Origin	Release date	SR reaction	YR reaction	Farmers’ rating*
Danda'a	EIAR-CIMMYT	2010	Moderately resistant	Moderately resistant	Very good to excellent
Digelu	EIAR-CIMMYT	2005	Susceptible	Susceptible	Very poor
Hidassie	EIAR-CIMMYT	2012	Susceptible	Moderately susceptible	Poor to good
Kingbird	EIAR-CIMMYT	2014	Moderately resistant	Moderately susceptible	Good to very good
Liben	EIAR-ICARDA	2015	Resistant	Moderately susceptible	Na
Shorima	EIAR-ICARDA	2011	Resistant	Susceptible	Excellent

*Disease and pest resistance rating by farmers^54^.

### Experimental design

The selected wheat varieties were planted on August 10, 2020, in two side-by-side blocks using a modified randomized complete block design (RCBD) with large plot sizes (5 m × 5 m), each with three randomized replicates, resulting in 36 plots (2 blocks × 6 varieties × 3 replicates). Due to heavy rains in July 2020, planting was delayed, which increased the probability of water scarcity towards the end of the main season. Before planting, the experimental area (0.15 ha) was leveled to obtain uniform and flat land.

The two blocks differed in their treatment with fungicides. One block was almost rust-free (disease controlled by fungicide applications, herein referred to as *treated*), representing the control group. The other block was rust-infected (without fungicide control, herein referred to as *non-treated*). To prevent fungicide drift between the two blocks, a strip of 4 m land was left unplanted. For each block, the experimental design consisted of three rows 2 m apart from each other, and each row had six plots 1.5 m apart from each other. Within each plot, the spacing between planted rows was 20 cm (Fig. 1).

Plots were fertilized with N-P-S at 121 kg/ha and UREA at 150 kg/ha following the recommended rates of the standard wheat crop nursery management at KARC. From the flowering stage to the maturity stage, irrigation was applied every afternoon for four hours. In the treated block, rust was controlled using the fungicide Rex^®^ Duo 497 SC (Epoxiconazole + Thiophanate-methyl; BASF, Ludwigshafen, Germany) at a rate of 0.5 l/ha, applied with handheld sprayers on September 25, 2020, and October 10, 2020. After the second application, fungicides were not further applied because the disease pressure was not favored by the climatic conditions. For yield measurements, a 4 m^2^ area per plot was harvested, and grain yield was measured in grams, with the grain moisture adjusted to 12% and converted to tonnes per hectare (t/ha).

For YR and SR disease increase, single spreader rows of bulk susceptible varieties were planted per plot using a mixture of varieties of varying degrees of SR and YR susceptibility: for SR PBW-343 and Hidassie and for YR Kubsa, Digelu, and Morocco. SR was inoculated with a bulk SR inoculum on September 25, 2020, with a ULV (Ultra Low Volume) sprayer and the injection method using a syringe on October 2, 2020, to ensure homogeneous infection development within the 5 m^2^ plots. A natural YR infection occurred across the experimental site because plots were planted late and surrounding earlier-planted farmer fields had already been naturally infected by YR.

### Disease scoring

Disease scoring was carried out three times throughout the experimental duration. The disease scoring was started when YR pressure was at 5% severity and SR pressure at 2% severity per plot. Subsequent scoring dates were chosen according to the disease development in the plots. Following standardized rust scoring guidelines^56–59^, five visual sub-scores were taken per plot, one in each corner and one in the center, capturing rust severity and the host plant response to each rust disease. For severity estimation, the Modified Cobb Scale^57^ was used, and the host plant response to infection was visually assessed according to disease symptoms^59^.

To obtain a representative rust scoring value per plot, the rust severity and host response data were combined by first calculating the coefficient of infection (CI) for each sub-score per scoring date and then averaging the resulting CI values across the plot per scoring date (ACI). The CI is calculated by multiplying the intensity of severity in percent with a host response constant, corresponding to a specific host response class^57^, using Eq. (1):

1 $$CI=D \times HR$$

where *CI* is the coefficient of infection in percent, *D* is the severity in percent, and *HR* is the host response constant according to specific host response class^57^.

In classical plant pathology studies, individual rust diseases are scored and treated separately. From an RS perspective, however, both YR and SR affect the plant canopy reflectance as a whole. Thus, for RS-based crop health measurements at the canopy level, an overall disease index (DI) needs to be computed to reflect the combined impact of both diseases on the canopy cover. Therefore, a DI was created to describe the severity and the host plant response to both rust diseases by merging the ACI of YR with the ACI of SR. From the literature, no specific method is recommended on how to merge multiple diseases into one DI, so the following two scenarios were tested (Eqs. 2 and 3):

Scenario X: a simple sum of YR and SR was calculated for each scoring date, whereby the weight coefficients for both diseases were set to 1 to represent an equal disease impact on the canopy cover.

2 $${DI}_{X}=1\times {ACI}_{YR}+1\times {ACI}_{SR}$$

where *DI*_*X*_ is the non-weighted disease index in percent and *ACI* is the averaged coefficient of infection per plot in percent for each *YR* and *SR* scoring date. This equation is applied to each disease scoring date equally.

Scenario W: a weighted sum of YR and SR was calculated with a reduced weight coefficient for SR according to the rust impact on plant parts relevant to photosynthesis. The rationale behind this is that the SR impact on relevant plant parts is delayed and consequently does not directly or indirectly affect green tissue by its first appearance. Thus, when SR appeared for the first time (in this study: DAS 80), its weight coefficient was reduced in the *DI* calculation, testing stepwise values from 0 (no SR impact) to 0.9, while it would increase to 1 at later stages.

3 $${DI}_{W}=1\times {ACI}_{YR}+wc\times {ACI}_{SR}$$

where *DI*_*W*_ is the weighted disease index in percent, *ACI* is the averaged coefficient of infection per plot in percent for each *YR* and *SR* scoring date, and *wc* is the weight coefficient for SR.

By calculating the *DI*_*X*_ and *DI*_*W*_ for each disease scoring date, DI time series were computed for both scenarios. To ensure that each scoring variable contributes equally to the time series and that DI time series would exhibit the same range of disease infection (e.g., not exceeding 100%), the DI values were normalized to the maximum and minimum observed DI across the entire time series following a min–max normalization approach. This data normalization was applied to both DI scenarios, resulting in DI scoring dates having normalized DI (DI_norm_) values expressed between 0 and 1.

### Remote sensing—UAV and satellite

To analyze the potential of UAV and VHRS-based multispectral sensors, very high spatial and temporal resolutions were needed, considering the plot size of the experiment and the dense cloud cover during the *Meher* season. Table 2 shows the characteristics of the tested RS sensors.

**Table 2:** Characteristics of RS sensors.

Platform	Sensor	Spectral wavelengths (Bands) [nm]^a^	Spatial resolution [m]^b^
UAV	Sequoia	530–570 (G) 640–680 (R) 730–740 (RE) 770–810 (NIR)	0.05
VHRS	Pleiades	430–550 (B) 500–620 (G) 590–710 (R) 740–940 (NIR) 470–830 (PAN)	0.5 (PAN) 2 (MS)
	SkySat	450–515 (B) 515–595 (G) 605–695 (R) 740–900 (NIR) 450–900 (PAN)	0.57–0.86 (PAN) 0.75–1.0 (MS)

^a^B: Blue; G: Green; R: Red; RE: Red-Edge; NIR: near-infrared; PAN: panchromatic.

^b^MS: multispectral.

**Figure 2:**
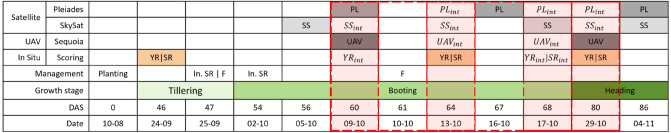
Overview of the data collection timeline (red dashed line: temporal focus window; light red box: sampling date; In. SR: inoculation of SR; F: fungicide application; DAS: day after sowing; YR: yellow rust; SR: stem rust; PL: Pleiades; SS: SkySat; UAV: unmanned aerial vehicle; int: interpolated).

UAV data acquisition was carried out at the canopy level using the quadcopter UAV platform Parrot Bluegrass Fields equipped with the on-board multispectral Parrot Sequoia sensor weighing < 2 kg (Parrot Drones S.A.S, Paris, France). The nominal radio link range of the platform was 2 km with a maximum flight time of 25 min. The Sequoia sensor provided spectral images, covering the electromagnetic spectrum from green to near-infrared (Table 2). Targeting the booting and heading wheat growth stages, the UAV was flown twice 60 m above ground within the temporal window of solar noon ± 2 h in clear sky conditions, covering an area of ~ 1 ha with the experimental site in its center (Fig. 2). Images were acquired with 80% front and 80% side overlap, resulting in a spatial ground resolution of 5.6 cm. Before each flight, radiometric sensor calibrations and corrections were performed using the standard reflectance calibration panel provided by the manufacturer. In addition, the built-in sunshine sensor measured the sun irradiance during each flight, enabling radiometric correction of images taken under distinct light conditions. Photogrammetric postprocessing was applied to the multispectral images using Pix4Dmapper software (v4.6.4; Pix4D, Lausanne, Switzerland). Following the standard processing pipeline (e.g., image alignment, tie point extraction, bundle block adjustment, point cloud densification), the images were converted into georeferenced, geometrically corrected and radiometrically calibrated reflectance maps and resampled to 5 cm ground resolution.

To address the spatial and temporal resolution needed for this study, two state-of-the-art VHRS constellations were selected: the SkySat (Planet Labs, San Francisco, CA, United States) and Pleiades (French Space Agency, Paris, France) satellite systems. Due to their sensor specifications (Table 2), both enable the acquisition of high probability cloud-free imagery at the sub-meter pixel level over a very frequently cloudy region.

Based on the CubeSat/nano-satellite concept, the SkySat constellation includes 21 high-resolution earth imaging satellites capable of sub-daily (up to 6–7 times per day on worldwide average) recordings of panchromatic and multispectral images at spatial resolutions of 0.57–0.86 m and 0.75–1.0, respectively, depending on the satellite generation. The panchromatic image is a single-band grayscale image covering the spectral range of 450–900 nm. The multispectral image consists of four multispectral bands (B, G, R, and NIR). Through ESA’s Third Party Missions program, three orthorectified, radiometrically calibrated and georeferenced SkySat scenes were obtained (Fig. 2). The satellite data were delivered as a 4-band multispectral SkySat Ortho Analytic Surface Reflectance product, already pan-sharpened to 0.5 m and corrected to bottom-of-atmosphere reflectance. Pan-sharpening refers to the fusion of a higher spatial resolution panchromatic image with a lower spatial resolution multispectral image^60,61^.

The Pleiades High-Resolution Optical Imaging Constellation provides optical high-resolution panchromatic and multispectral satellite imagery with daily coverage due to the identical Pleiades satellite twins (Pleiades 1A and Pleiades 1B). The panchromatic data present a spatial resolution of 0.50 m, and the spectral range is 470–830 nm, while multispectral data present a spatial resolution of 2.00 m and include four multispectral bands (B, G, R, and NIR). Three orthorectified (Ortho Level 3), radiometrically corrected, and georeferenced Pleiades scenes were acquired from Airbus Defence and Space Intelligence (Toulouse, France) (Fig. 2). These scenes were delivered as panchromatic-multispectral ortho products, corrected from atmospheric systematic contributions (static effects), resulting in top-of-atmosphere reflectance. The top-of-atmosphere reflectance values can be directly assimilated to bottom-of-atmosphere reflectance if imagery was taken in clear sky conditions^62^, which was our case. To enhance the spatial resolution of the multispectral information, all Pleiades datasets were pan-sharpened using the Gram-Schmidt pan-sharpening method^63^ with Pleiades-specific multispectral band weights.

Subsequently, all UAV and VHRS datasets were manually co-registered to six ground control points (GCPs) installed at the experimental site (one GCP at each corner and two GCPs in the gap between the two treatment blocks) using ArcGIS Desktop 10.8.1 (ESRI Inc., Redlands, CA, United States). The spectral G, R, and NIR bands that all three sensors have in common were selected to derive wheat disease and crop health-related VIs for each RS dataset. Those spectral bands can be considered YR-sensitive bands^10,42,44,45^, while the NIR band is able to increase the spectral response of YR by reducing possible noise (e.g., atmospheric effects)^42^. In total, 15 different VIs were computed. Table 3 depicts all 18 spectral features used in this study.

**Table 3:** Overview of the spectral bands and VIs used.

Index	Formula		Traits	Relevant studies
Spectral bands				
Green (G)	${R}_{G}$		Disease (YR)	^10,42,44,45^
Red (R)	${R}_{R}$		Disease (YR)	^10,42,44,45^
Near-infrared (NIR)	${R}_{NIR}$			^42^
VIs				
Chlorophyll index—green (CIG)	$\left(\frac{{R}_{NIR}}{{R}_{G}}\right)-1$		Chlorophyll	^64^
Chlorophyll vegetation index (CVI)	$\frac{({R}_{NIR}*{R}_{R})}{{{R}_{G}}^{2}}$		Chlorophyll	^64^
Green normalized difference vegetation index (GNDVI)	$\frac{({R}_{NIR}-{R}_{G})}{({R}_{NIR}+{R}_{G})}$		Disease (YR), chlorophyll, LAI, nitrogen, water content	^10,42,43,45^
Modified soil adjusted vegetation index (MSAVI)	${0.5[2R}_{NIR}+1-\sqrt{{{(2R}_{NIR}+1)}^{2}-8\left({R}_{NIR}-{R}_{R}\right)}$]		Disease (YR)	^45^
Modified simple ratio (MSR)	$\frac{(\frac{{R}_{NIR}}{{R}_{R}}-1)}{\sqrt{(\frac{{R}_{NIR}}{{R}_{R}})+1}}$		Disease	^46^
Normalized difference vegetation index (NDVI)	$\frac{({R}_{NIR}-{R}_{R})}{({R}_{NIR}+{R}_{R})}$		Disease (YR), chlorophyll, LAI, biomass, yield	^10,42–45^
Normalized green red difference index (NGRDI)	$\frac{({R}_{G}-{R}_{R})}{({R}_{G}+{R}_{R})}$		Chlorophyll, biomass, water content	^64^
Optimized soil-adjusted vegetation index (OSAVI)	$\frac{({R}_{NIR}-{R}_{R})}{({R}_{NIR}+{R}_{R}+0.16)}$		Disease	^46^
Ratio index Red/Green (RGR)	$\frac{{R}_{R}}{{R}_{G}}$		Disease (YR)	^10,43,44^
Ratio vegetation index (RVI)	$\frac{{R}_{R}}{{R}_{NIR}}$			
Re-normalized difference vegetation index (RDVI)	$\frac{({R}_{NIR}-{R}_{R})}{\sqrt{({R}_{NIR}+{R}_{R})}}$		Disease (YR)	^10^
Soil adjusted vegetation index (SAVI)	$\frac{({1+L)*(R}_{NIR}-{R}_{R})}{({R}_{NIR}+{R}_{R}+\mathrm{L})}$	L = 0.5	Disease (YR)	^10,43,45^
Simple ratio (SR)	$\frac{{R}_{NIR}}{{R}_{R}}$		Disease, biomass, water content, nitrogen	^46^
Triangular vegetation index (TVI)	$0.5[120({R}_{NIR}-{R}_{G})-200({R}_{R}-{R}_{G})]$		Disease (YR), green LAI, chlorophyll, canopy	^42,43,45^
Visible atmospherically resistant index (VARI_G_)	$\frac{({R}_{G}-{R}_{R})}{({R}_{G}+{R}_{R})}$		Disease (YR)	^10,43,44^

*R*: measured reflectance at the original wavelength or wavebands specified by the subscript (G: Green; R: Red; NIR: near-infrared).

### Data analysis

UAV and VHRS datasets were acquired over the experimental site, and ground truthing in the form of visual disease scoring was carried out. Figure 2 shows the data collection timeline, revealing data gaps for both RS and in situ data, i.e., missing RS data on scoring dates or vice versa. Due to unexpected factors (e.g., COVID-19 travel restrictions, weather conditions), data could not be collected in equal time steps.

To overcome this challenge, a twenty-day temporal focus window from days after sowing (DAS) 60 until DAS 80 (2020-10-09 until 2020-10-29) with four specific sampling dates was established by using the first and last UAV flights. The temporal window covers wheat development during the booting and heading stages. Linear interpolation was applied to postprocessed scoring, UAV and VHRS spectral feature datasets to harmonize sampling dates (DAS 60, DAS 64, DAS 68, and DAS 80), enabling extraction of both real measured and interpolated values from each data source.

Subsequently, disease data were analyzed using the area under the disease progress curve (AUDPC) with the trapezoidal method, i.e., Riemann's integrals^65^. RS-derived spectral features from both UAV and VHRS sensors were extracted as the mean per plot (1 m buffer to avoid mixed pixels from spreader rows and paths), and the area under the curve (AUC) was computed for the individual wavelengths and VIs using Riemann's integrals. These calculations allow for integrating the temporal information from disease scores and all three RS datasets into single variables (AUDPC and AUC). Using Pearson correlation analysis, the relationship between disease scoring (DI_norm_) and spectral features per sampling date was investigated to evaluate the early detection potential of specific RS sensors. The data from AUDPC and AUCs were assessed in terms of their individual association with each other and with grain yield also through Pearson correlation analysis. Spectral features and AUCs having at least a highly significant correlation (r > 0.8; r < − 0.8; *p* ≤ 0.05) with DI_norm_, AUDPC, and yield, respectively, were selected to assess the prediction potential of RS derivates based on a linear regression model, expressed through the adjusted coefficient of determination (R^2^_adj_) as corrected goodness-of-fit and the root mean square error (RMSE) as model quality measures. The data processing and analyses were implemented in the statistical software R, version 4.1.3^66^.

## Results

### Temporal development of rusts

The visual disease scoring results revealed that early natural YR infection took place, and SR inoculation was effective (Table 4). YR was present in the plots on all scoring dates, and SR was present only on the last scoring date. The first YR scoring (DAS 46) shows that YR infected all varieties, even in the treated block. This resulted from a delay in fungicide application, as the first fungicide application was carried out at DAS 47. The data confirm the effectiveness of the fungicide application, as observed in the considerable YR infection drop in the treated block recorded on DAS 64. Overall, the YR scorings underpin the expected YR variety responses according to their known susceptibility to YR; e.g., Digelu reacted quickly and strongly to YR, as it is the most susceptible variety. The late appearance of SR around heading and grain filling is typical^57^. Of all varieties, Hidassie was highly susceptible to SR, as expected. The high susceptibility of Digelu to early YR infections (50–70 S at DAS 64) meant that plants were largely dead before SR developed.

**Table 4:** Visual disease scoring results as plot averages.

ID	Variety	Block^a^	Visual scores for YR^b^			Visual scores for SR^b^		
			DAS 46	DAS 64	DAS 80	DAS 46	DAS 64	DAS 80
1	HIDASSIE	T	5MS	5MR	5MR	0	0	10S
2	DIGELU		30S	15S	15S	0	0	5S
3	DANDA'A		5MS	tMR	tMR	0	0	5MS
4	KINGBIRD		15S	5MR	5MR	0	0	5MS
5	LIBEN		15S	tMR	tMR	0	0	tMS
6	SHORIMA		5MS	5MR	5MR	0	0	tMS
7	DANDA'A	NT	5MS	5MR	10MS	0	0	5MS
8	HIDASSIE		5MS	20S	30S	0	0	40S
9	DIGELU		30S	60S	90S	0	0	5S
10	SHORIMA		5MS	10S	60S	0	0	5S
11	LIBEN		15S	15MS	40S	0	0	5MSS
12	KINGBIRD		15S	10MSS	20SMS	0	0	5MS
13	SHORIMA		5MS	10MS	30SMS	0	0	tMS
14	DANDA'A		5MS	5MS	tMS	0	0	5MS
15	KINGBIRD		10MS	5MS	5MS	0	0	5MS
16	LIBEN		15S	tMS	30SMS	0	0	5MS
17	HIDASSIE		5MS	10S	10MSS	0	0	40S
18	DIGELU		30S	50S	80S	0	0	tS
19	DIGELU	T	30S	tMR	tMR	0	0	5S
20	DANDA'A		5MS	tMR	tMR	0	0	5S
21	HIDASSIE		5MS	5MR	5MR	0	0	15S
22	SHORIMA		10MS	tMR	tMR	0	0	tS
23	LIBEN		5MS	0	0	0	0	tS
24	KINGBIRD		10MS	tMR	tMR	0	0	tS
26	HIDASSIE		5MS	10MR	10MR	0	0	10S
27	LIBEN		10MS	tMR	tMR	0	0	tS
28	KINGBIRD		tMS	tMR	tMR	0	0	5S
29	DIGELU		20S	tMR	tMR	0	0	5S
30	SHORIMA		5MS	5MR	5MR	0	0	tS
31	DANDA'A	NT	5MS	5MS	tMS	0	0	tS
32	HIDASSIE		5MS	10S	30S	0	0	50S
33	SHORIMA		5MS	10MSS	20SMS	0	0	tS
34	DIGELU		30S	70S	90S	0	0	5S
35	KINGBIRD		10MS	10S	5MS	0	0	5MSS
36	LIBEN		10MS	20S	40S	0	0	5S

^a^T: TREATED, fungicide treatment block; NT: NON-TREATED, non-fungicide treatment block.

^b^S: susceptible; MSS: moderately susceptible to susceptible; MS: moderately susceptible; MR: moderately resistant; t: trace level of severity^59^.

**Figure 3:**
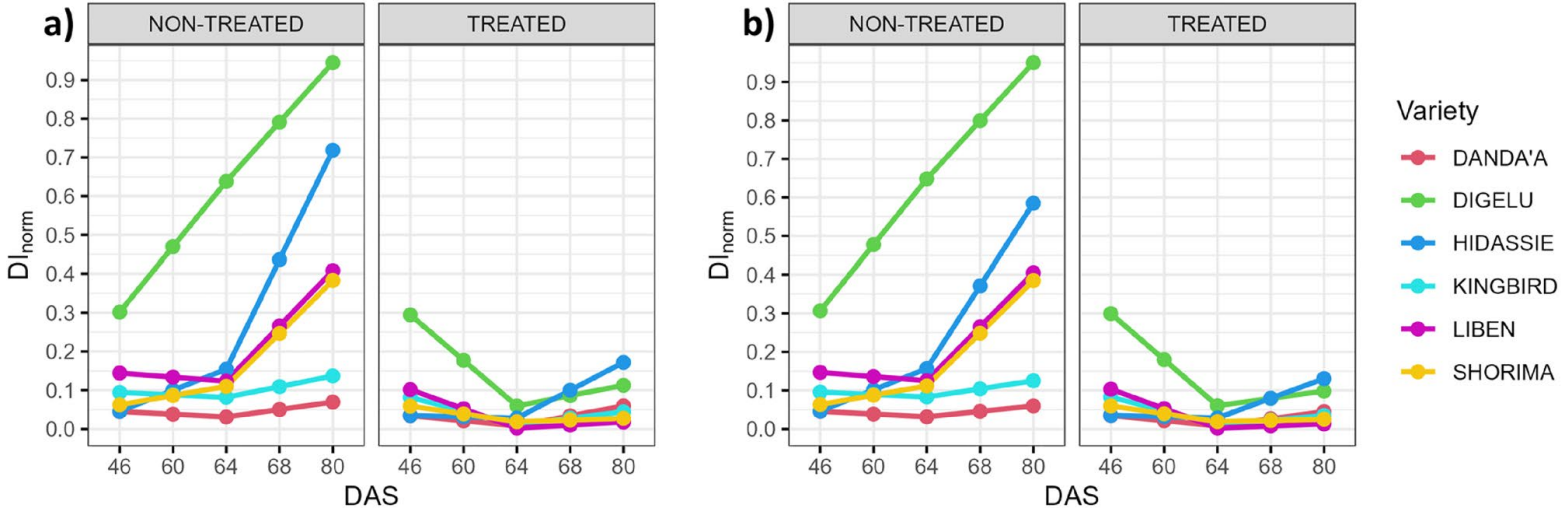
Disease progression per wheat variety and treatment block for disease merging scenarios—(**a**) Scenario X (simple sum) and (**b**) Scenario W_0.7_ (weighted sum).

Figure 3 shows the interpolated disease progression curves for the different disease index (DI_norm_) merging scenarios—Scenario X (simple sum) and Scenario W (weighted sum) (here: using Scenario W_0.7_ based on the weight coefficient 0.7 as example). Focusing on the non-treated block, both scenarios reveal similar patterns of variety response to disease presence, confirming the high YR susceptibility of Digelu and high SR susceptibility of Hidassie, respectively, followed by moderate YR susceptibility of Liben and Shorima and relatively high YR and SR resistance of Kingbird and Danda’a. The main difference between both scenarios was observed in the SR response of Hidassie. When SR was scored in the plots for the first time (DAS 80), Scenario X revealed a stronger response to SR with a high increment (DAS 64 to DAS 80) of 0.57 (0.15 to 0.72) compared to Scenario W_0.7_ with an increment of 0.42 (0.16 to 0.58).

### Effect of rusts on yield

**Figure 4:**
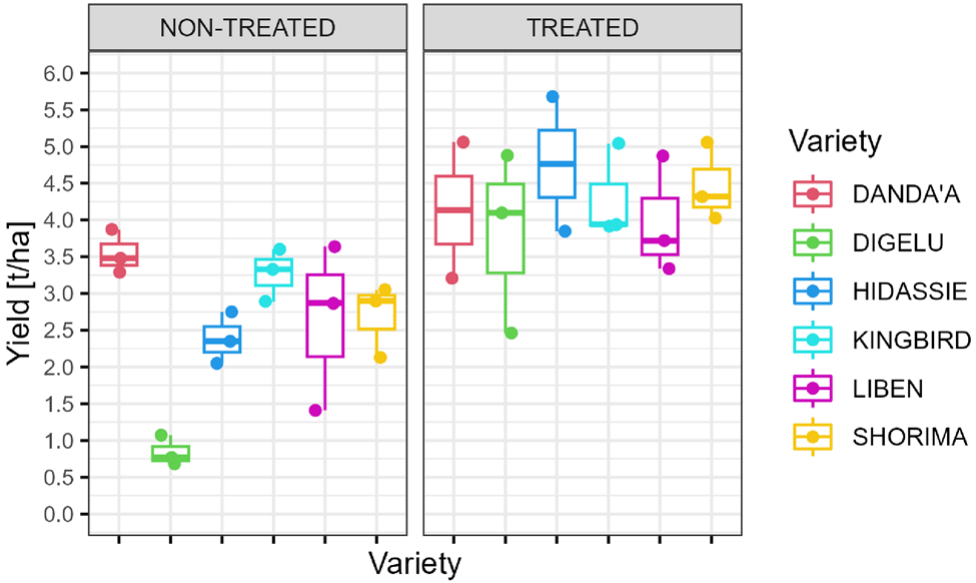
Box plot showing recorded yield per variety and treatment block.

Overall, a statistically significant (*p* < 0.001) difference in yield production was found between the treated and non-treated blocks (mean: 4.2 t/ha vs. 2.6 t/ha). Plot yield records show a relatively low variation across varieties for the treated block, while for the non-treated block, higher variation exists (variance: 0.7 t/ha vs.1 t/ha), with minimum yield for more susceptible varieties such as Digelu (0.8 t/ha) and Hidassie (2.4 t/ha) and maximum yield for more resistant varieties such as Danda’a (3.6 t/ha) and Kingbird (3.3 t/ha) (Fig. 4).

Comparing the yield performance of individual varieties between the two treatments (fungicide vs. non-fungicide) showed that the yield of all varieties was decreased by rusts within the non-treated plots. Yield loss can be associated with the degree of rust resistance/susceptibility of each variety. The highest yield loss was observed in the most susceptible varieties (Digelu: 78%; Hidassie: 50%), while the lowest yield loss was observed in the most resistant variety (Danda’a: 14%) (Table 5).

**Table 5:** Grain yield per variety and yield loss caused by rust disease calculated as the percentage difference in grain yield (t/ha) between fungicide and non-fungicide treatments.

Variety	Non-treated			Treated			Yield loss [%]
	AUDPC_X_^a^	AUDPC_W_^b^	Yield [t/ha]	AUDPC_X_^a^	AUDPC_W_^b^	Yield [t/ha]	
Danda'a	1.02	0.93	3.55	0.70	0.55	4.14	14
Digelu	15.49	15.64	0.84	1.96	1.83	3.81	78
Hidassie	8.62	7.31	2.38	2.00	1.60	4.77	50
Kingbird	2.20	2.09	3.27	0.62	0.53	4.30	24
Liben	5.34	5.32	2.64	0.30	0.26	3.98	34
Shorima	4.88	4.92	2.69	0.50	0.49	4.47	40

Due to the delay in fungicide treatment, incomplete control for YR was obtained in treated plots, meaning that yield losses between treatments would likely have been higher under complete control.

^a^AUDPC_X_: AUDPC for Scenario X.

^b^AUDPC_W_: AUDPC for Scenario W_0.7._

**Figure 5:**
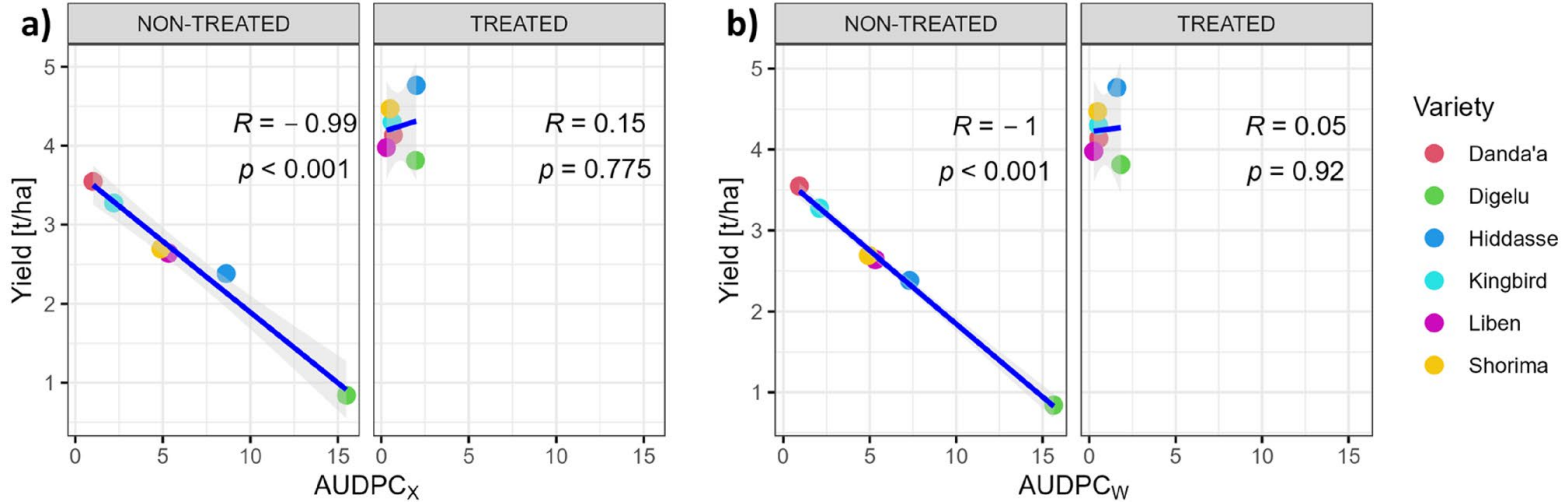
Correlation between grain yield and AUDPC—(**a**) Scenario X (simple sum) and (**b**) Scenario W_0.7_ (weighted sum) (AUDPC_X_: AUDPC for Scenario X; AUDPC_W_: AUDPC for Scenario W_0.7_).

For Scenario X and Scenario W (using the example of Scenario W_0.7_), Fig. 5 shows the correlation analysis of yield with the AUDPC, revealing a very high negative and significant (*p* ≤ 0.001) relationship for non-treated plots (Scenario X: r = − 0.99; Scenario W_0.7_: r = − 1). This result clearly illustrates the anticipated variety reaction to SR and YR, already considered during the variety selection process. As expected, the correlation between AUDPC and yield in the treated block was negligible (Scenario X: r = 0.15; Scenario W_0.7_: r = 0.05).

### Remote sensing

To investigate the potential of UAV and VHRS for rust disease detection and phenotyping, Scenario W was used as an example. The results belonging to Scenario X are presented in the Supplementary Data.

### Rust detection

To assess the capability of multispectral UAV and VHRS sensors for rust detection at the variety level, the relationships between the visual disease scoring (DI_norm_) and spectral features derived from UAV and VHRS across all different varieties were statistically evaluated in the non-treated block for each sampling date. Using Pearson correlation analysis, Tables 6 and 7 summarize the relationships at DAS 60 (booting stage) and at DAS 80 (heading stage), respectively.

**Table 6:** Pearson correlation and linear regression analysis between DI_norm_ and spectral features at DAS 60 (non-fungicide treatment block) (note: the results are the same for both scenarios).

	UAV			SkySat			Pleiades		
	r	R^2^_adj_	RMSE	r	R^2^_adj_	RMSE	r	R^2^_adj_	RMSE
G	0.94**	0.86**	0.05	0.90*	0.75*	0.06	0.91*	0.79*	0.06
R	0.91*	0.78*	0.06	0.90*	0.77*	0.06	0.90*	0.76*	0.06
NIR	0.85*	0.65*	0.08	0.80			0.83*	0.61*	0.08
NGRDI	0.26			0.39			− 0.32		
NDVI	− 0.46			− 0.51			− 0.01		
GNDVI	− 0.78			− 0.76			0.17		
TVI	0.66			0.44			0.69		
CVI	− 0.71			− 0.70			0.49		
CIG	− 0.83*	0.61*	0.08	− 0.73			0.18		
RGR	− 0.27			− 0.36			0.32		
RDVI	0.30			0.06			0.56		
VARIg	0.26			0.39			− 0.32		
OSAVI	0.03			− 0.51			− 0.01		
MSR	− 0.5			− 0.45			0		
MSAVI2	0.41			− 0.53			− 0.01		
RVI	0.44			0.53			0.01		
SAVI	0.38			− 0.51			− 0.01		
SR	− 0.52			− 0.42			0		

*p-value < 0.05; **p-value < 0.01; and ***p-value < 0.001.

**Table 7:** Scenario W_0.7_ – Pearson correlation and linear regression analysis between DI_norm_ and spectral features at DAS 80 (non-fungicide treatment block) (results for Scenario X are presented in Supplementary Data S Table 1).

	UAV			SkySat			Pleiades		
	r	R^2^_adj_	RMSE	r	R^2^_adj_	RMSE	r	R^2^_adj_	RMSE
G	0.51			0.59			0.44		
R	0.77			0.82*	0.59*	0.17	0.73		
NIR	− 0.61			− 0.27			− 0.19		
NGRDI	− 0.84*	0.63*	0.16	− 0.79			− 0.96**	0.90**	0.08
NDVI	− 0.93**	0.83**	0.11	− 0.98***	0.95***	0.06	− 0.99***	0.97***	0.05
GNDVI	− 0.86*	0.68*	0.15	− 0.85*	0.66*	0.16	− 0.92**	0.81**	0.12
TVI	− 0.89*	0.75*	0.13	− 0.92**	0.81**	0.12	− 0.83*	0.62*	0.16
CVI	− 0.43			− 0.36			0.08		
CIG	− 0.86*	0.68*	0.15	− 0.83*	0.61*	0.17	− 0.93**	0.82**	0.11
RGR	0.85*	0.65*	0.16	0.79			0.96**	0.90**	0.09
RDVI	− 0.92**	0.81**	0.12	− 0.98***	0.96***	0.05	− 0.91*	0.79*	0.12
VARIg	− 0.84*	0.63*	0.16	− 0.79			− 0.96**	0.90**	0.08
OSAVI	− 0.93**	0.83**	0.11	− 0.98***	0.95***	0.06	− 0.99***	0.97***	0.05
MSR	− 0.92**	0.81**	0.12	− 0.97**	0.93**	0.07	− 0.99***	0.97***	0.05
MSAVI2	− 0.90*	0.77*	0.13	− 0.99***	0.96***	0.05	− 0.99***	0.97***	0.05
RVI	0.93**	0.84**	0.11	0.99***	0.96***	0.05	0.99***	0.97***	0.05
SAVI	− 0.91*	0.79*	0.12	− 0.98***	0.95***	0.06	− 0.99***	0.97***	0.05
SR	− 0.91*	0.79*	0.12	− 0.96**	0.91**	0.08	− 0.99***	0.97***	0.05

*p-value < 0.05; **p-value < 0.01; and ***p-value < 0.001.

Across all three sensors, the Pearson coefficient revealed that the visible G and R bands were very highly significantly positively correlated with the DI_norm_ at DAS 60 (r ≥ 0.9; *p* ≤ 0.05) (Table 6). To a minor degree, the NIR bands derived from UAV (r = 0.85) and Pleiades (r = 0.83) as well as the chlorophyll-related CIG derived from UAV (r = − 0.83) showed a highly significant correlation. The positive correlation of the G and R bands with DI_norm_ suggests that the higher the spectral reflectance is, the higher the disease pressure in the corresponding variety. At DAS 60, only YR was present in the plots with low DI_norm_ values of 0.04–0.14, except for the highly susceptible Digelu with a DI_norm_ of 0.48.

**Figure 6:**
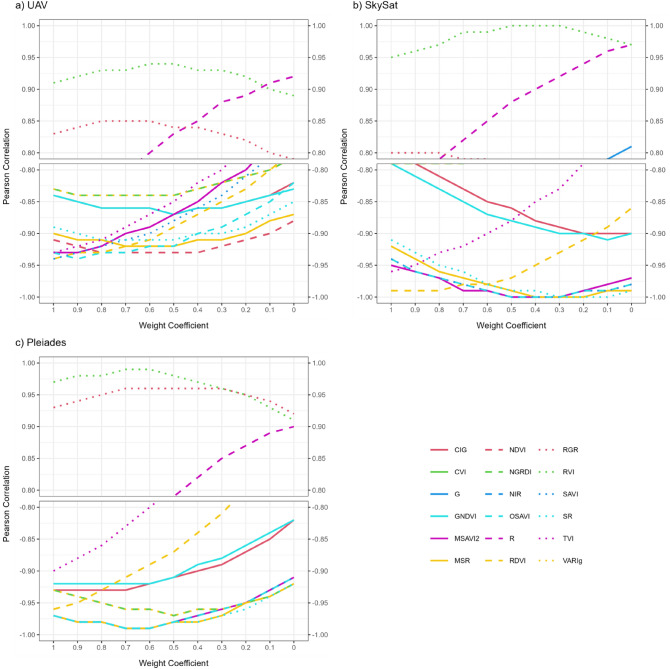
Scenario W with tested weight coefficients – Pearson correlation between DI_norm_ and spectral features derived from (**a**) UAV, (**b**) SkySat, and (**c**) Pleiades at DAS 80 (non-fungicide treatment block) (note: Scenario W_1.0_ with the weight coefficient of 1.0 is the same as Scenario X; Scenario W_0_ with the weight coefficient of 0 excludes the SR impact).

For Scenario W at DAS 80, Fig. 6 provides an overview of the impact of tested weight coefficients on the relationships between DI_norm_ and spectral features. Focusing on strong and significant correlations (r > 0.81; r < − 0.81; *p* ≤ 0.05), the correlation strength varied slightly (± 0.01) for multiple spectral features from UAV using the weight coefficient 0.9, 0.8, and 0.7, SkySat using 0.5 to 0.1, and Pleiades using 0.9 to 0.5. Across all sensors, weight coefficients 0.7, 0.6, and 0.5 yielded more often strongest correlations. Subsequently, Scenario W_0.7_ based on the weight coefficient 0.7 was used as an example to demonstrate the RS rust detection and HTP capabilities in more detail.

For Scenario W_0.7_ at DAS 80, highly significant positive and negative relationships (r ≥ 0.9; r ≤ − 0.9) were found, yielding the best significant correlations with NDVI, OSAVI, RVI, RDVI, and MSR for the UAV-based sensor (*p*  ≤ 0.01); MSAVI2, RVI, NDVI, RDVI, OSAVI, and SAVI for SkySat (*p* ≤ 0.001); and NDVI, OSAVI, MSR, MSAVI2, RVI, SAVI, and SR for Pleiades (*p* ≤ 0.001) (Table 7). Moreover, a general decrease in the relevance of visible G and R bands was observed, whereby for the G band only low to moderate non-significant correlation (r: 0.44–0.59) and for R bands still high (partly significant) correlations (r: 0.73–0.82) were identified. At DAS 80, both YR and SR rust diseases were scored in the plots with low DI_norm_ values of 0.06 and 0.13 for Danda’a and Kingbird, moderate DI_norm_ values of 0.38 and 0.40 for Shorima and Liben, and relatively high DI_norm_ values of 0.58 and 0.95 for Hidassie and Digelu.

**Figure 7:**
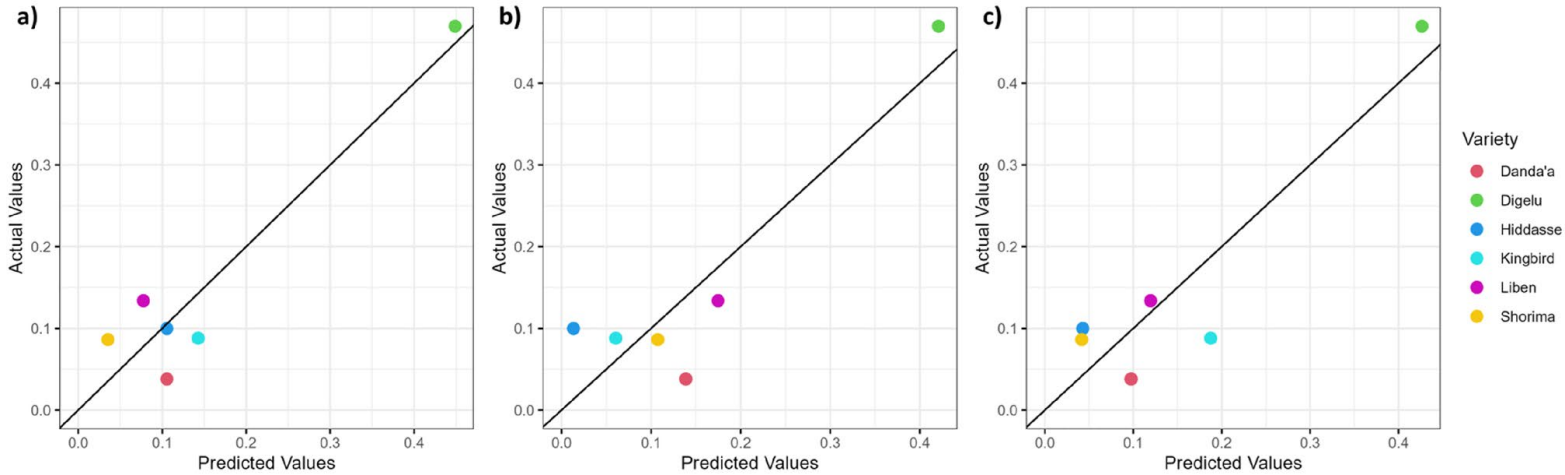
Predicted versus measured DI_norm_ values at DAS 60 based on the regression equation of the linear model using (**a**) G derived from UAV, (**b**) R derived from SkySat, and (**c**) G derived from Pleiades data (black line: one-to-one line) (note: the results are the same for both scenarios).

**Figure 8:**
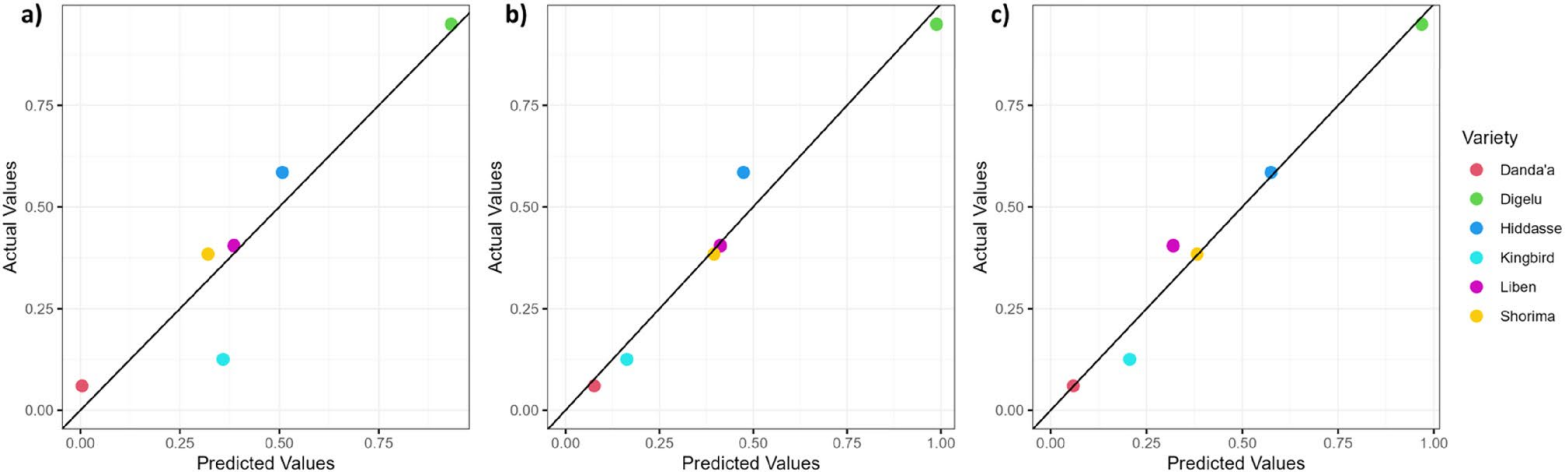
Scenario W_0.7_—Predicted versus measured DI_norm_ values at DAS 80 based on the regression equation of the linear model using (**a**) RVI derived from UAV, (**b**) RVI derived from SkySat, and (**c**) RVI derived from Pleiades data (black line: one-to-one line) (results for Scenario X are presented in Supplementary Data S Fig. 1).

The regression analysis of spectral features highly correlated (r > 0.8; r < − 0.8; *p* ≤ 0.05) with visual disease scoring indicated that remotely sensed data from all three sensors are highly suitable to estimate disease scoring in the field at early crop growth stages (Tables 6 and 7). For the booting stage (DAS 60), the single G bands followed by the R bands derived from UAV (R^2^_adj_ = 0.86; R^2^_adj_ = 0.78), SkySat (R^2^_adj_ = 0.75; R^2^_adj_ = 0.77), and Pleiades (R^2^_adj_ = 0.79; R^2^_adj_ = 0.76) imagery were identified as the best (significant) predictor variables for DI_norm_, and to a moderate degree, the NIR bands from UAV (R^2^_adj_ = 0.65) and Pleiades (R^2^_adj_ = 0.61) as well as CIG from UAV (R^2^_adj_ = 0.61). For the heading stage (DAS 80), a wide range of VIs were identified with high (R^2^_adj_ ≥ 0.7) to very high (R^2^_adj_ ≥ 0.9) explanatory power for DI_norm_. For UAV imagery, RVI (R^2^_adj_ = 0.84), NDVI (R^2^_adj_ = 0.83), and OSAVI (R^2^_adj_ = 0.83) were the top 3 performing significant predictors (*p* ≤ 0.01), while for VHRS imagery, the five VIs MSAVI2, RVI, NDVI, OSAVI, and SAVI, (SkySat: R^2^_adj_ ≥ 0.95; Pleiades: R^2^_adj_ = 0.97) as well as for only SkySat RDVI (R^2^_adj_ = 0.96) and for only Pleiades MSR and SR (R^2^_adj_ = 0.97) performed very highly at the highest significance level (*p* ≤ 0.001). At a lower significance level (*p* ≤ 0.01), other very high performing VIs were MSR and SR (R^2^_adj_ = 0.93; R^2^_adj_ = 0.91) for SkySat or NGRDI, RGR, and VARIg (R^2^_adj_ = 0.9) for Pleiades. From the single bands, only the R band from SkySat showed moderate explanatory power (R^2^_adj_ = 0.59). Figures 7 and 8 depict the relationships between DI_norm_ and the top-performing spectral features for each RS sensor at the booting and heading stages, respectively. It is clearly shown that the predictive power at DAS 60 depends on the highly susceptible Digelu variety.

### High-throughput phenotyping

Analysis of the relationships of the AUCs of individual bands and VIs with the AUDPC_W_ (Scenario W_0.7_) and yield across early crop growth stages (from booting to heading) revealed that a wide range of spectral features derived from UAV imagery had high to very high significant correlations. However, only one spectral feature from SkySat imagery was highly significantly correlated with disease development (AUDPC_W_) and yield under the non-fungicide treatment (r > 0.8; r < − 0.8; *p* ≤ 0.05) (Tables 8 and 9). Under the non-fungicide treatment (non-treated block), the Pearson coefficient (*r*) of the interactions between the single spectral bands and AUDPC_W_ was 0.81 and 0.91 for UAV G and R bands as well as 0.83 for SkySat R band. The correlations of VIs and AUDPC_W_ were -0.85 to -0.92 (MSR, GNDVI, CIG, and NDVI) and 0.94 (RVI) for UAV, while VHRS-derived VIs were not significantly correlated to AUDPC_W_ (Table 8). From all individual bands, the Pearson coefficient revealed a significant relationship with yields of -0.92 and -0.84 (*p* ≤ 0.05) only for the AUCs from the UAV and SkySat R bands, respectively, under non-fungicide treatment. Moreover, only five VIs derived from UAV imagery showed a significant (*p* ≤ 0.05) correlation with yield (r: 0.82–0.91; GNDVI, CIG, MSR, and NDVI; r = − 0.93 RVI), while VI-AUCs from VHRS were not significant (*p* > 0.05) (Table 9). The data suggest that the varieties with higher G-AUCs, R-AUCs, and/or RVI-AUCs also had higher disease pressure in terms of AUDPC (positive correlation) and lower yields (negative correlation), while varieties with higher NDVI-AUCs, GNDVI-AUCs, CIG-AUCs, and/or MSR-AUCs had lower disease pressure in terms of AUDPC (negative correlation) but higher yields (positive correlations) (Table 5).

**Table 8:** Scenario W_0.7_ – Pearson correlation and linear regression analysis between AUDPC_W_ and the AUC of spectral features (non-fungicide treatment block) (results for Scenario X are presented in Supplementary Data S Table 2).

	UAV			SkySat			Pleiades		
	r	R^2^_adj_	RMSE	r	R^2^_adj_	RMSE	r	R^2^_adj_	RMSE
G-AUC	0.81*	0.57*	2.79	0.73			0.72		
R-AUC	0.91*	0.80*	1.93	0.83*	0.62*	2.64	0.79		
NIR-AUC	0.54			0.58			0.59		
NGRDI-AUC	− 0.19			− 0.01			− 0.78		
NDVI-AUC	− 0.92**	0.81**	1.85	− 0.71			− 0.59		
GNDVI-AUC	− 0.85*	0.65*	2.52	− 0.68			− 0.30		
TVI-AUC	0.01			− 0.01			0.30		
CVI-AUC	− 0.58			− 0.49			0.43		
CIG-AUC	− 0.85*	0.66*	2.50	− 0.64			− 0.29		
RGR-AUC	0.26			0.05			0.80		
RDVI-AUC	− 0.58			− 0.38			0.09		
VARIg-AUC	− 0.19			− 0.01			− 0.78		
OSAVI-AUC	− 0.77			− 0.71			− 0.59		
MSR-AUC	− 0.85*	0.65*	2.54	− 0.63			− 0.55		
MSAVI2-AUC	− 0.39			− 0.73			− 0.61		
RVI-AUC	0.94**	0.85**	1.63	0.73			0.61		
SAVI-AUC	− 0.49			− 0.71			− 0.59		
SR-AUC	− 0.80			− 0.60			− 0.53		

*p-value < 0.05; **p-value < 0.01; and ***p-value < 0.001.

**Table 9:** Pearson correlation and linear regression analysis between grain yield and the AUC of spectral features (non-fungicide treatment block).

	UAV			SkySat			Pleiades		
	r	R^2^_adj_	RMSE	r	R^2^_adj_	RMSE	r	R^2^_adj_	RMSE
G-AUC	− 0.80			− 0.73			− 0.72		
R-AUC	− 0.92*	0.80*	0.35	− 0.84*	0.64*	0.47	− 0.79		
NIR-AUC	− 0.55			− 0.58			− 0.60		
NGRDI-AUC	0.22			0.04			0.78		
NDVI-AUC	0.91*	0.79*	0.35	0.71			0.58		
GNDVI-AUC	0.82*	0.60*	0.49	0.67			0.28		
TVI-AUC	− 0.02			0.01			− 0.31		
CVI-AUC	0.55			0.46			− 0.47		
CIG-AUC	0.83*	0.61*	0.48	0.62			0.27		
RGR-AUC	− 0.29			− 0.08			− 0.81		
RDVI-AUC	0.57			0.37			− 0.10		
VARIg-AUC	0.22			0.04			0.78		
OSAVI-AUC	0.76			0.71			0.58		
MSR-AUC	0.84*	0.63*	0.47	0.64			0.54		
MSAVI2-AUC	0.37			0.73			0.60		
RVI-AUC	− 0.93**	0.83**	0.32	− 0.73			− 0.60		
SAVI-AUC	0.47			0.71			0.58		
SR-AUC	0.80			0.60			0.52		

*p-value < 0.05; **p-value < 0.01; and ***p-value < 0.001.

While the relationships for various spectral bands and VIs with yield were noticeably stronger under the non-fungicide treatment, these relationships were—as expected—not significant under the fungicide treatment, except for the SkySat R band (r = − 0.84; *p* ≤ 0.05). This is explained by the almost complete absence of the disease, i.e., photosynthesis, and thus yield, were unaffected in non-diseased plants. However, as a natural YR infection occurred before the first fungicide application, all varieties showed slight disease symptoms (DI_norm_: 0.03–0.10) on DAS 46, except Digelu (DI_norm_: 0.29). After two fungicide applications on DAS 47 and DAS 61, the DI_norm_ dropped to ≤ 0.03 for all varieties and to 0.06 for Digelu, as recorded on DAS 64. Thus, the data revealed that single NIR bands and several VIs from VHRS (SkySat: TVI and RDVI; Pleiades: GNDVI, TVI, CIG, RDVI) showed significant (*p* ≤ 0.05) strong positive correlations of ≥ 0.9 and ≥ 0.83, respectively, with AUDPC_W_ but were not shown for UAV data.

**Figure 9:**
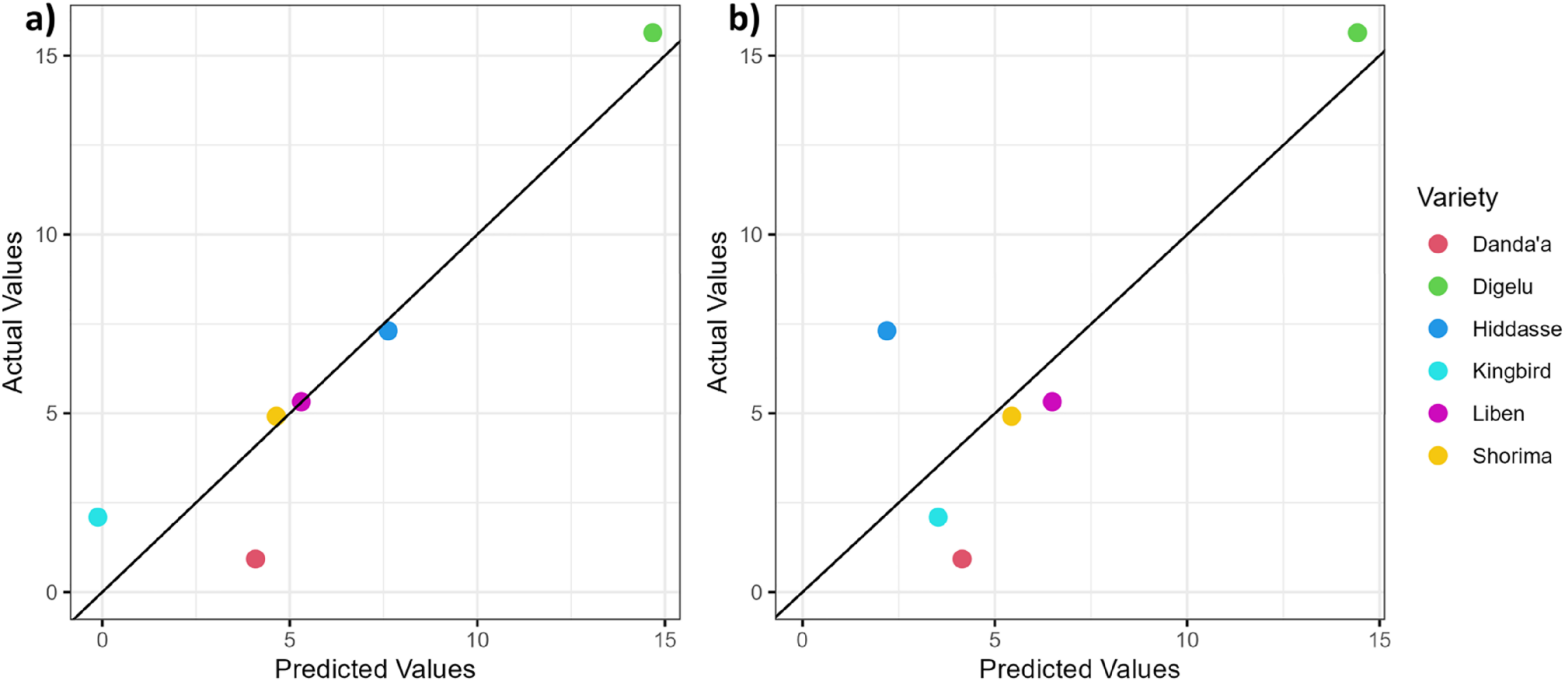
Scenario W_0.7_—Predicted versus measured AUDPC_W_ values based on the regression equation of the linear model using (**a**) RVI-AUC derived from UAV and (**b**) R-AUC derived from SkySat data (black line: one-to-one line) (results for Scenario X are presented in Supplementary Data S Fig. 2).

**Figure 10:**
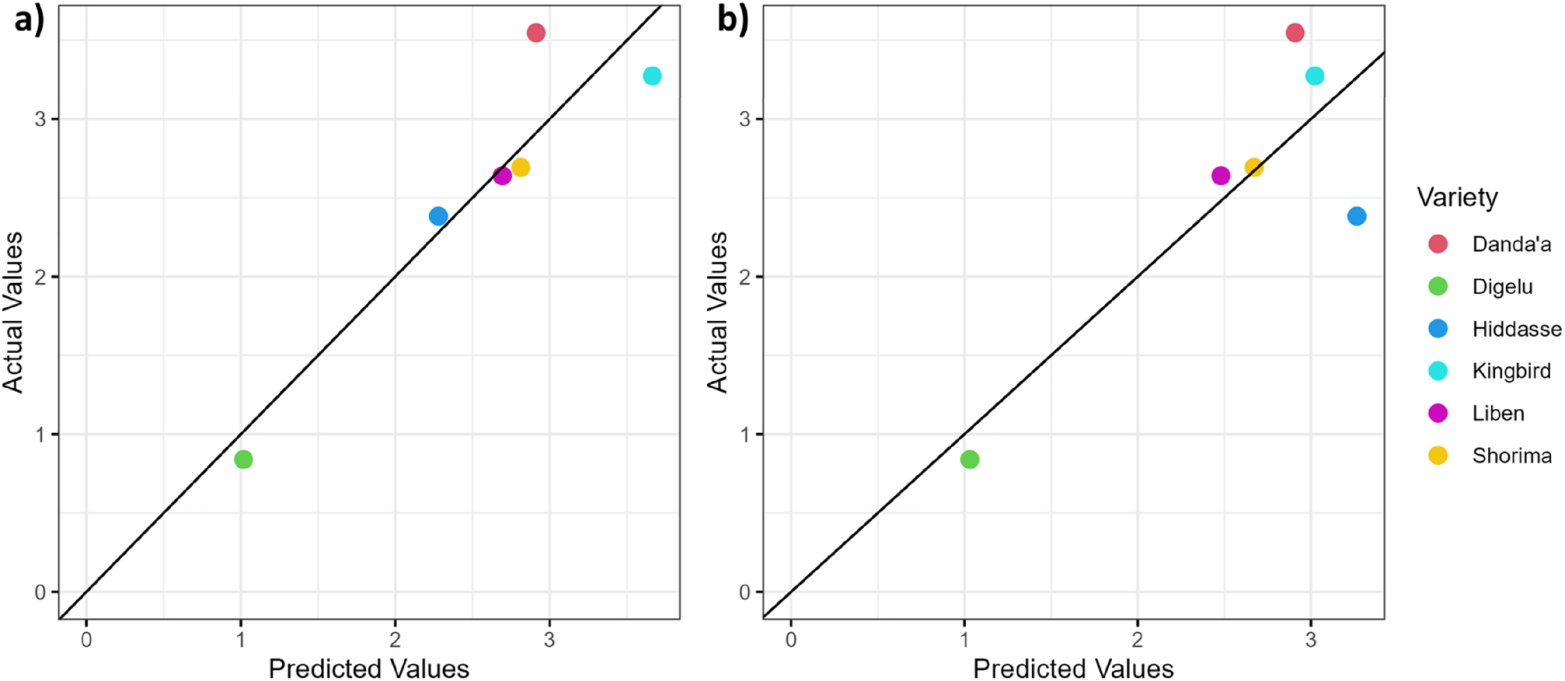
Predicted versus measured grain yield values based on the regression equation of the linear model using (**a**) RVI-AUC derived from UAV and (**b**) R-AUC derived from SkySat data (black line: one-to-one line).

To assess the prediction potential of remotely sensed spectral features to estimate varieties’ response to disease stress and grain yield, only AUCs having at least a high significant correlation (r > 0.8; r < − 0.8; *p* ≤ 0.05) with AUDPC_W_ or yield were explored using linear regression analysis (Tables 8 and 9). From all individual bands, the results revealed that R-AUC is the best (significant) predictor variable for AUDPC_W_ and grain yield across UAV (AUDPC_W_: R^2^_adj_ = 0.8; yield: R^2^_adj_ = 0.8) and SkySat (AUDPC_W_: R^2^_adj_ = 0.62; yield: R^2^_adj_ = 0.64) sensors. Only the UAV-derived VI-AUCs RVI and NDVI were found to have high explanatory power for AUDPC_W_ (R^2^_adj_ = 0.85 and R^2^_adj_ = 0.81, respectively) and yield (R^2^_adj_ = 0.83 and R^2^_adj_ = 0.79, respectively), whereas CIG, MSR, and GNDVI showed moderate power for AUDPC_W_ (R^2^_adj_: 0.65–0.66) and yield (R^2^_adj_: 0.6–0.63). VI-AUCs derived from VHRS were not significant. Figures 9 and 10 depict the relationships of AUDPC_W_ and grain yield (respectively) with top-performing spectral features for each RS sensor.

## Discussion

To the best of the authors’ knowledge, the present study is the first to explore RS capabilities from both UAV and satellite platforms for phenotyping and detecting multiple wheat rust diseases in the same plot.

A specific method for integrating multiple rust diseases has not yet been suggested in the existing literature. Commonly, literature exploring RS for wheat rust disease detection, monitoring, and forecasting focuses on identifying a specific disease (binary classification; e.g., disease vs. healthy)^10,42,44,45^, differentiating among multiple diseases or a specific disease from other stresses such as pest or abiotic stress (multicategory classification; e.g., disease1 vs. disease2 vs. healthy, disease vs. pest vs. healthy; disease vs. abiotic stress vs. healthy)^43^, and retrieving discrete or continuous infection/severity levels of a specific disease (quantification/regression; e.g., severe, moderate, slight disease vs. healthy)^44^. To obtain a representative ground observation that describes the disease impact on the canopy cover as a whole, two scenarios for merging different visual disease scores from multiple rust diseases in wheat were proposed according to rust pathology expert knowledge: Scenario X (a simple sum) and Scenario W (a weighted sum). Scenario X represents an equal impact of both diseases, while for Scenario W, weight coefficients for SR from 0 (no SR impact) to 0.9 were stepwise tested, considering the disease-specific temporal processes leading to a direct or indirect alternation of photosynthesis-relevant plant parts and consequently canopy cover. Results indicate that optimal weight coefficients depend on spectral sensor resolution and vary for different spectral features. For disease detection, coefficients 0.7, 0.6, and 0.5 yielded stronger correlations across all sensors more often. Subsequently, Scenario W_0.7_ based on the weight coefficient 0.7 was used. YR infections occur primarily on leaves and head, producing symptoms such as yellowing and necrosis of leaves as soon as infection takes place. In contrast, primary SR infections are typically on the stem with no or little impact on the canopy. As SR infection progresses, the disease starts damaging the xylem tissue of the plant that translocate water and dissolved minerals from the roots to the rest of the plant, leading to a reduction of nutrient flow to the leaves and weakening of the stem^67^. This process eventually leads to a reduction in photosynthesis and finally leaf death (i.e., canopy effects), revealing a temporal delay of observable canopy impact from infection to occurrence. The scenario approach reported here represents a first step to addressing multiple disease pressures. The weighted coefficient for SR is an attempt to align the biological process of pathogen infection and impacts on canopy level parameters relevant for the remote sensing in a rapid and easily applicable way. Detailed experiments measuring canopy photosynthetic changes solely with SR infections may result in more refined coefficient in the future. However, for RS applications such as crop disease detection and monitoring, more research on how to address the simultaneous occurrence of multiple diseases in the same plot is needed, as this is the most likely situation in farmer fields. In particular, the question of how to integrate different disease scoring results associated with different diseases into one representative ground observation, considering the different rates of disease development, needs to be addressed to enable the overall crop health condition at the canopy level related to biotic stresses to be determined.

In terms of rust progression, both scenarios revealed realistic overall scoring results. For example, the impact of the later-appearing SR disease on the SR-susceptible variety Hidassie showed an increase of 57% (Scenario X) and 42% (Scenario W_0.7_) within 16 days from no to first SR appearance, which is found within the range of reported rates of SR development (e.g., up to 80% within 2–3 weeks)^6^. The reduced values for Hidassie of Scenario W_0.7_ reflect the delayed reaction on photosynthesis-relevant plant parts during SR infection.

Our results suggest that potential yield losses from rust disease in improved bread wheat may be as high as 78% in susceptible varieties under strong disease pressure. This number seems to be realistic, as up to 100% yield loss for both YR^68^ and SR^69^ diseases had been reported. The very strong correlation of AUDPC_W_ or AUDPC_X_ with grain yield (r ≥ − 0.99) confirms the overall impact that wheat rusts can exert on wheat production following a severe epidemic in susceptible wheat varieties, i.e., historic YR epidemics in Ethiopia (1977–1990) resulted in yield losses from 30 to 96% at various spatial scales^70–72^.

The application of multispectral and hyperspectral proximal and satellite RS for disease resistance phenotyping and disease monitoring has been extensively discussed in the literature for a wide range of crops^25,36,40,41,73–77^. A recent review focusing on YR and LR detection and monitoring in wheat^41^ reported moderate to very high accuracies when using field spectrometers (71–97%), UAV/aircraft-based multispectral and hyperspectral imaging systems (> 60–91%), and multispectral satellite imagery (57–90%).

Our results underpin the potential use of UAV and VHRS multispectral imaging for rapid wheat rust disease detection and phenotyping, both less time-consuming and less prone to human error than traditional disease scoring and field surveys as well as less expensive than hyperspectral imaging systems. VHRS RS is able to bridge the gap from plot scale to regional scale applications, whereby VHRS is advantageous over UAV by not requiring large initial investments for purchasing the RS systems and trained specialists for data acquisition and processing, and it has fewer technical challenges^29,78^.

For various multispectral satellite sensors (e.g., GeoEye, Ikonos-2, Quickbird, RapidEye, SPOT-5 and -6, WorldView-2, and Sentinel-2), several previous studies on wheat YR mapping demonstrated—by using RS imagery and in situ data around the grain filling stage (post flowering/anthesis)—that the most spectrally sensitive and highly significant reflectance bands can be found mainly in the red region of the electromagnetic spectrum, followed by the green and near-infrared regions^10,42–45^. Moreover, the authors found crop growth-related VIs such as NDVI, TVI, GNDVI, SAVI, RGR, VARIg, and EVI to be significantly sensitive to wheat rusts, applying them successfully for identifying YR^10,44,45^, differentiating between YR and powdery mildew diseases^43^, and determining YR infection/severity levels^44^.

In contrast, our study focused on the late vegetative stage of the wheat life cycle, in particular the booting and heading stages, which are the predecessors of the flowering (anthesis) and grain filling stages. A focus on earlier wheat growth stages is highly desirable to improve the prevention of disease outbreaks and epidemics. The earlier the crop stage at which rust infection starts, the more damage will occur. However, earlier crop growth stages have not been considered in satellite-based rust-detection and monitoring studies before.

For both combined disease score scenarios, our study revealed that depending on the time of sensing, the G band was correlated best among all tested 18 spectral features and slightly better than the R band (except for SkySat) at the booting stage, while for the heading stage, the correlation power of G and R bands was reduced, being non-significant for the G and R bands, except for the SkySat R band of Scenario W_0.7_. As YR alters plant leaf color from green to yellow, the G band was highly sensitive to detect YR at the booting stage (Fig. 7). The results confirmed the critical need for including highly susceptible varieties in on-station field experiments and disease screening nurseries as reference points for control checks regarding disease occurrence and development. As disease pressure increased in the non-treated plots over time, a clear shift in the spectral sensitivity to wheat rust was observed from solely visible G and R spectral bands at the booting stage to multispectral ratio VIs at the heading stage, taking advantage of the NIR band in combination mostly with the R band (e.g., RVI, NDVI, MSR, OSAVI, RDVI, MSAVI2, SAVI, and SR) and to a lesser extent with the G band (e.g., GNDVI and CIG). This observation algins with identified significant spectral ranges between YR-infected and non-infected wheat at both leaf and canopy levels^77^.

Our results identified a large set of VIs significantly sensitive to wheat rusts and consequently suitable for early detection and monitoring, supporting the findings of previous studies^10,43–45^. Thus, the very highly correlated spectral features identified in our study (Tables 6 and 7; S1) exhibited for all three sensors a high (R^2^_adj_ ≥ 0.7) to very high (R^2^^2^_adj_ ≥ 0.9) explanatory power to estimate disease scoring values at earlier growth stages using linear regression models. The most relevant predictive spectral features at the booting stage were the G band (UAV, Pleiades) and R band (SkySat), and those at the heading stage were RVI, NDVI, OSAVI, MSR, RDVI (UAV) and NDVI, OSAVI, MSAVI2, RVI, SAVI (SySat, Pleiades), RDVI (SkySat), SR (Pleiades). In contrast to Yuan et al.^42^, a higher coefficient of determination was found in our study for NDVI and GNDVI.

For phenotyping applications, our study showed that several spectral features derived or calculated from UAV and VHRS multispectral imagery were highly correlated (r > 0.8; r < − 0.8; *p* ≤ 0.05) with disease progression and/or grain yield under non-fungicide treatments (Tables 8 and 9; S3). The strongest relationships with grain yield were found for UAV with RVI, R band, and NDVI and for SkySat with the R band. Strong relationships between AUDPC and R bands (UAV and SkySat) and RVI and NDVI (UAV) indicate the potential usage of these spectral features as an auxiliary tool for disease phenotyping and are potentially suitable for forecasting yield losses caused by wheat rusts. It must be noted that both VHRS sensors were weaker predictors than UAV due to the lack of sensitivity of VHRS multispectral instruments in terms of spectral resolution (broad spectral bands) and spatial resolution.

This study was designed as a proof of concept to demonstrate UAV and VHRS remote sensing capabilities for assessing the variety response to disease stress. To satisfy the need of a representative sample of spectral satellite information per plot (pixels per plot), a compromise between large field plots and many field plots had to be made due to limited available land and resources. Fewer larger field plots than commonly used in agricultural trials were used, resulting in a minimum set of 18 data points, aggregated to 6 at the variety level. Whilst meeting the absolute minimum for statistical tests used, this relatively small number has limitations in terms of interpretability. Although further research is needed, the methodology presented in this study is a starting point tackling the initial capital investment constraint faced by research stations when it comes to HTP techniques for disease resistant genotypes selection, which also allows for multi-location genotype trials.

## Conclusion

The current study identifies several spectral features from UAV and VHRS multispectral imagery that have strong assessment power for the detection of combined wheat rust diseases at early crop growth stages. Novel approaches to account for the simultaneous occurrence of multiple rust diseases were tested. Visible spectral (VIS) bands (G, R) were more useful at early stages (booting), shifting to VIS–NIR VIs at later stages (heading). This study provides valuable insight into the upscaling capability of multispectral sensors for disease detection from UAV imagery at 5 cm per pixel to pan-sharpened satellite imagery at 50 cm per pixel, demonstrating a first step towards upscaling disease detection from plot to regional scales. Further work will expand and improve current methodology to examine the VHRS detection capability towards machine and deep learning techniques (e.g., convolutional neural network) to allow for continuous monitoring systems, focusing on both single and mixed rust diseases under different treatments (e.g., variable fungicide rates, irrigation rates).

### Supplementary Information

Supplementary Information.

## Data Availability

Remote sensing data from SkySat were provided through the European Space Agency Third Party Mission scheme (ESA TPM) and Pleiades by the Université Catholique de Louvain. All other data that support the findings of this study are available from the corresponding author upon reasonable request.
