# Supplementary material for: The potential of UAV and very high-resolution satellite imagery for yellow and stem rust detection and phenotyping in Ethiopia
Source: Sci Rep. 2023 Oct 5;13:16768. doi: 10.1038/s41598-023-43770-y (PMC10556098; doi:10.1038/s41598-023-43770-y)
Supplement: Supplementary file 1 — Supplementary Information. [file 41598_2023_43770_MOESM1_ESM.docx]

**The potential of UAV and very high-resolution satellite imagery for yellow and stem rust detection and phenotyping in Ethiopia**

Gerald Blasch^1*^, Tadesse Anberbir^2^, Tamirat Negash^3^, Lidiya Tilahun^3^, Fikrte Yirga Belayineh^3^, Yoseph Alemayehu^1^, Girma Mamo^2^, David P. Hodson^4^, Francelino A. Rodrigues Jr.^4,5^

**Supplementary Data**

S Table 1. Scenario X – Pearson correlation and linear regression analysis between DI_norm_ and spectral features at DAS 80 (non-fungicide treatment block).

|  | UAV | | | SkySat | | | Pleiades | | |
| --- | --- | --- | --- | --- | --- | --- | --- | --- | --- |
|  | r | R^2^_adj_ | RMSE | r | R^2^_adj_ | RMSE | r | R^2^_adj_ | RMSE |
| G | 0.38 |  |  | 0.48 |  |  | 0.31 |  |  |
| R | 0.68 |  |  | 0.72 |  |  | 0.63 |  |  |
| NIR | -0.73 |  |  | -0.40 |  |  | -0.33 |  |  |
| NGRDI | -0.83* | 0.61* | 0.17 | -0.79 |  |  | -0.93** | 0.83** | 0.11 |
| NDVI | -0.91* | 0.78* | 0.13 | -0.94** | 0.85** | 0.10 | -0.97** | 0.93** | 0.07 |
| GNDVI | -0.84* | 0.62* | 0.17 | -0.79 |  |  | -0.92** | 0.81** | 0.12 |
| TVI | -0.93** | 0.84** | 0.11 | -0.96** | 0.89** | 0.09 | -0.90* | 0.77* | 0.13 |
| CVI | -0.40 |  |  | -0.29 |  |  | 0.03 |  |  |
| CIG | -0.84* | 0.63* | 0.17 | -0.76 |  |  | -0.93** | 0.82** | 0.12 |
| RGR | 0.83* | 0.62* | 0.17 | 0.80 |  |  | 0.93** | 0.83** | 0.11 |
| RDVI | -0.94** | 0.85** | 0.11 | -0.99*** | 0.97*** | 0.05 | -0.96** | 0.90** | 0.09 |
| VARIg | -0.83* | 0.61* | 0.17 | -0.79 |  |  | -0.93** | 0.83** | 0.11 |
| OSAVI | -0.93** | 0.84** | 0.11 | -0.94** | 0.85** | 0.10 | -0.97** | 0.93** | 0.07 |
| MSR | -0.90* | 0.76* | 0.13 | -0.92** | 0.81** | 0.12 | -0.97** | 0.93** | 0.07 |
| MSAVI2 | -0.93** | 0.84** | 0.11 | -0.95** | 0.87** | 0.10 | -0.97** | 0.93** | 0.07 |
| RVI | 0.91* | 0.78* | 0.13 | 0.95** | 0.87** | 0.10 | 0.97** | 0.93** | 0.07 |
| SAVI | -0.94** | 0.85** | 0.11 | -0.94** | 0.85** | 0.10 | -0.97** | 0.93** | 0.07 |
| SR | -0.89* | 0.74* | 0.14 | -0.91* | 0.78* | 0.13 | -0.97** | 0.92** | 0.08 |

* p-value < 0.05; ** p-value < 0.01; and *** p-value < 0.001


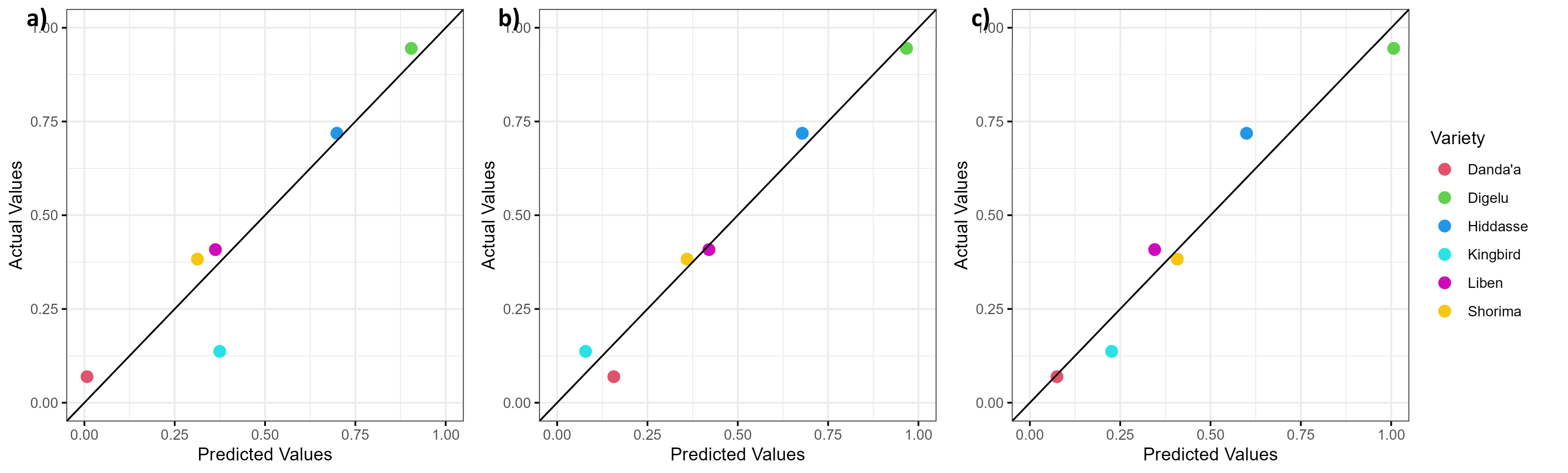


S Figure 1. Scenario X – Predicted versus measured DI_norm_ values at DAS 80 based on the regression equation of the linear model using **a)** RDVI derived from UAV, **b)** RDVI derived from SkySat, and **c)** NDVI derived from Pleiades data (black line: one-to-one line).

S Table 2. Scenario X – Pearson correlation and linear regression analysis between AUDPC_X_ and the AUC of spectral features (non-fungicide treatment block).

|  | UAV | | | SkySat | | | Pleiades | | |
| --- | --- | --- | --- | --- | --- | --- | --- | --- | --- |
|  | r | R^2^_adj_ | RMSE | r | R^2^_adj_ | RMSE | r | R^2^_adj_ | RMSE |
| G-AUC | 0.77 |  |  | 0.68 |  |  | 0.66 |  |  |
| R-AUC | 0.87* | 0.70* | 2.34 | 0.78 |  |  | 0.73 |  |  |
| NIR-AUC | 0.47 |  |  | 0.54 |  |  | 0.54 |  |  |
| NGRDI-AUC | -0.19 |  |  | -0.01 |  |  | -0.73 |  |  |
| NDVI-AUC | -0.92** | 0.80** | 1.90 | -0.66 |  |  | -0.56 |  |  |
| GNDVI-AUC | -0.84* | 0.64* | 2.58 | -0.63 |  |  | -0.30 |  |  |
| TVI-AUC | -0.05 |  |  | 0 |  |  | 0.26 |  |  |
| CVI-AUC | -0.58 |  |  | -0.44 |  |  | 0.40 |  |  |
| CIG-AUC | -0.84* | 0.63* | 2.62 | -0.58 |  |  | -0.28 |  |  |
| RGR-AUC | 0.26 |  |  | 0.05 |  |  | 0.75 |  |  |
| RDVI-AUC | -0.62 |  |  | -0.35 |  |  | 0.06 |  |  |
| VARIg-AUC | -0.19 |  |  | -0.01 |  |  | -0.73 |  |  |
| OSAVI-AUC | -0.79 |  |  | -0.66 |  |  | -0.56 |  |  |
| MSR-AUC | -0.83* | 0.61* | 2.68 | -0.58 |  |  | -0.52 |  |  |
| MSAVI2-AUC | -0.43 |  |  | -0.68 |  |  | -0.59 |  |  |
| RVI-AUC | 0.94** | 0.85** | 1.64 | 0.68 |  |  | 0.59 |  |  |
| SAVI-AUC | -0.53 |  |  | -0.66 |  |  | -0.56 |  |  |
| SR-AUC | -0.78 |  |  | -0.54 |  |  | -0.50 |  |  |

* p-value < 0.05; ** p-value < 0.01; and *** p-value < 0.001


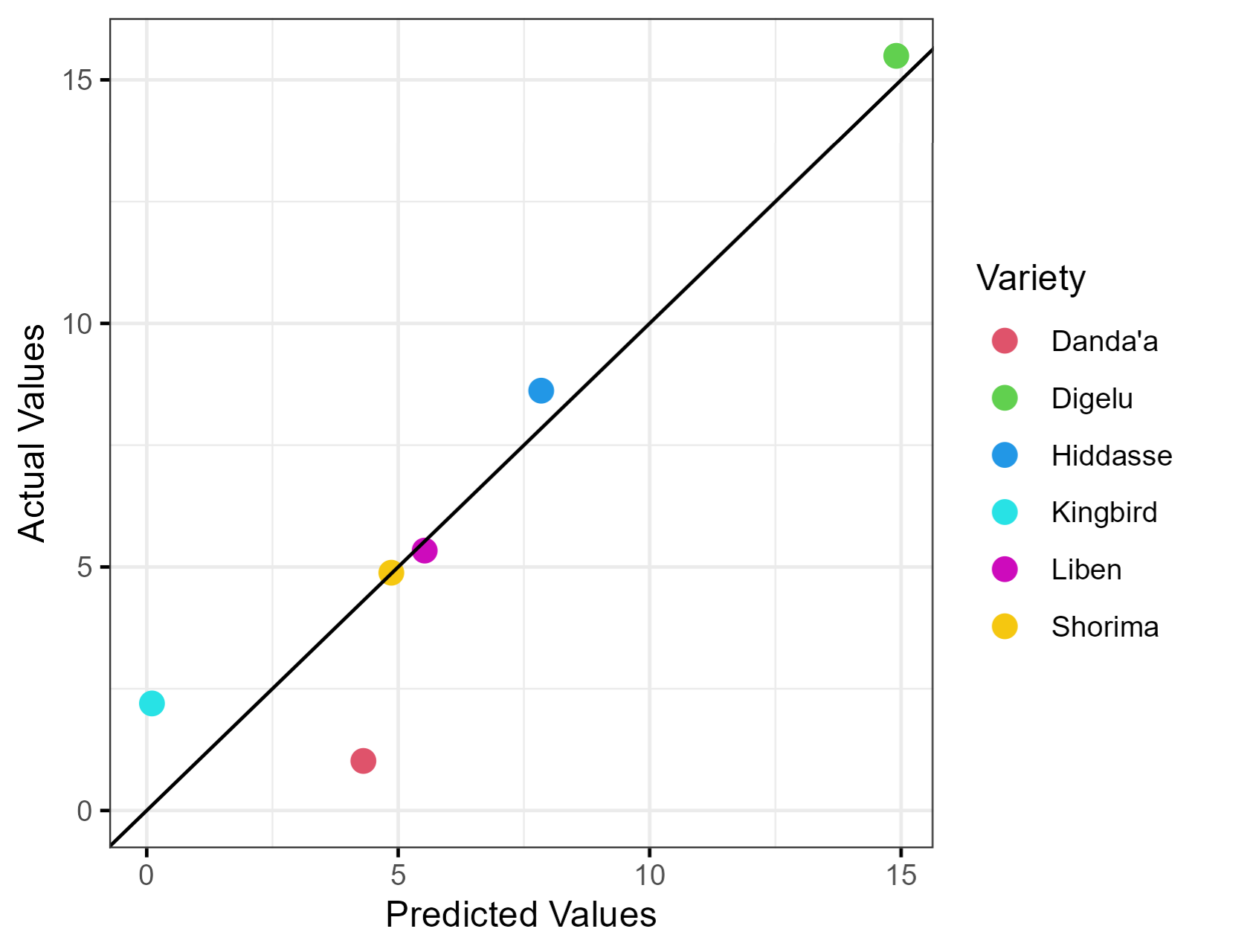


S Figure 2. Scenario X – Predicted versus measured AUDPC_X_ values based on the regression equation of the linear model using RVI-AUC derived from UAV data (black line: one-to-one line).
